# Enhanced killing of HepG2 during cryosurgery with Fe_3_O_4_-nanoparticle improved intracellular ice formation and cell dehydration

**DOI:** 10.18632/oncotarget.21499

**Published:** 2017-10-05

**Authors:** Fuquan Yuan, Gang Zhao, Fazil Panhwar

**Affiliations:** ^1^ Center for Biomedical Engineering, Department of Electronic Science and Technology, University of Science and Technology of China, Hefei 230027, Anhui, China; ^2^ Anhui Provincial Engineering Research Center for Biopreservation and Artificial Organs, Hefei 230027, Anhui, China

**Keywords:** cryosurgery, Fe_3_O_4_ nanoparticles, intracellular ice formation, cell dehydration, killing efficiency

## Abstract

Cryosurgery is a minimally invasive treatment that utilize extreme low temperatures to destroy abnormal tissues. The clinical monitoring methods for cryosurgery are almost based on the visualization of the iceball. However, for a normal cryosurgery process, the effective killing region is always smaller than the iceball. As a result, the end of the cryosurgery process can only be judged by the surgeons according to their experience. The subjective judgement is one of the main reasons for poor estimation of tumor ablation, and it sparks high probability of recurrence and metastasis associate with cryosurgery. Being different from the previous optimization studies, we develop a novel approach with the aid of nanoparticles to enlarge the effective killing region of entire iceball, and thus it greatly decrease the difficulty of precise judgement of the cryosurgery only by applying the common clinical imaging methods. To verify this approach, both the experiments on a tissue-scale phantom with embedded living HepG2 cells in agarose and on a cell-scale cryo-microscopic freeze-thaw stage are performed. The results indicate that the introduction of the self-synthesized Fe_3_O_4_ nanoparticles significantly improved cell killing in the cryosurgery and the range of killing is extended to the entire iceball. The potential mechanism is further revealed by the cryo-microscopic experiments, which verifies the presence of Fe_3_O_4_ nanoparticles can significantly enhance the probability of intracellular ice formation and the cell dehydration during freezing hence it promote precise killing of the cells. These findings may further promote the widespread clinical application of modern cryosurgery.

## INTRODUCTION

Cancer is a leading health care problem of world and the primary cause of death in China. According to the statistic, liver cancer is the most commonly diagnosed cancer in the males younger than 65 years-of-age, which is major cause of cancer death [1]. In the US, cancer deaths have decreased by 23% since 1991, however mortality due to hepatic cancers is increasing which frame cancer is a leading cause of death in 21 states [2]. At this time, chemotherapy, radiotherapy, surgical resection and combinations of these approaches are standard therapies of cancer treatments however each method has limitations and none of them is completely cure cancer. We need better curative methods with limited or no side effects. Cryosurgery or cryoablation is a minimally invasive therapy, in which tumor tissue is frozen by direct spraying cryogen or circulating a cryogen inside a probe tip [3–5]. Although cryoablation was initially used to treat breast and uterine cancers, it has been expanded to treat skin, prostate, kidney, liver, lung and bone tumors [6–13], which attracted wide attention and gained acceptance in the surgical community [14, 15]. Compared with traditional surgical resection, cryosurgery is minimally invasive and offers various benefits such as, less pain, less bleeding, and less post-surgical complications [3]. Cryosurgery is also less expensive and requires only a short recovery time or hospital stay [16, 17].

However, cryosurgery is not a standard cancer treatment for all cancer types due to some limitations. A successful cryosurgery has two goals, one is to destroy the tumor tissues completely, and the other is to minimize cryoinjury to the surrounding healthy tissue [18], Owing to the advent of modern imaging technologies, iceball monitoring is used to reduce cell death of non-cancerous tissues. However, one certain problem in cryosurgery is the insufficient freezing effect in the edge of tumor tissue which leads to recurrence and metastasis of tumors. [19–22]. During cryosurgery, the temperature at the iceball edge is greater (less cooler) than the center, contributing to ineffective killing of cancer cells at tumor edges [23].

To address cryosurgical efficacy issues, nanoparticles can be used to promote freezing efficiency and enhance cell killing while minimizing cryoinjury to the normal tissue. Lv's group conducted a feasibility study for thermal protection using microencapsulated phase change micro/nanoparticles during cryosurgery [24]. Shenoi and colleagues studied nanoparticle preconditioning for improved thermal conductivity in tumors [25] and Liu's group initially proposed the concept of nano-cryosurgery, injecting MgO nanoparticles into tumor tissue to control temperature distribution, also control the size of frozen area, and adjust iceball formation [26–28]. Bischof's group used TNF-α-coated gold nanoparticles for delivering TNF-α preferentially to tumors in an *in vivo* model [29]. In addition, many other researchers conducted numerical investigations on the effect of nanoparticles on cryosurgery [27, 30, 31].

Most of the studies were performed at the macroscopic level, and the crucial problem is that freezing is insufficient for killing the cancer cells at the edges of iceball. The effective killing temperature of cryosurgery may vary from -20°C to -40°C, and previous studies have shown that the temperature needs to go 1 cm beyond tumor edge to ensure sufficient ablation [4, 32–35]. The effective killing region is always smaller than the iceball, but the temperature distribution inside the iceball is invisible during cryosurgery. As a result, the end of the cryosurgery process can only be judged by the surgeons according to their experience based on the visualization of the iceball. However, the tumor cells cannot be completely killed in the frozen region, it is impossible to judge the end of cryosurgery intuitively. Furthermore, the microscopic level of mechanisms for both freezing injury at the cell scale and enhanced killing effect for tumor cells by nanoparticles added cryoablation remain unclear. Microscopic observations are necessary since previous studies shows that the phenomenon of intracellular freezing is closely related to the cell damage, and has been proved for a long time [36–38]. During freezing, first ice crystals will form in extracellular solution, which may break the balance of intracellular and extracellular chemical potentials. As a result, cells may perceive severe osmotic injuries caused by the chemical potential difference between intra and extracellular solutions. Further, high concentrations of intra and extracellular solutions may cause “solution injury” [37, 39, 40]. Nevertheless, the above mentioned micro-scale mechanisms during cryosurgery has not yet been completely explored.

In this study, we developed a new nanoparticle-aided approach to enlarge the effective killing region to almost the entire iceball, and thus to greatly decrease the difficulty of precise judgement in the end of cryosurgery only by using the commonly used clinical imaging methods. This approach was further verified by both the cell- and tissue-scale experiments with living HepG2 cells.

## RESULTS

### Fe_3_O_4_ nanoparticle synthesis, characterization and cytotoxicity

**Figure 1:**
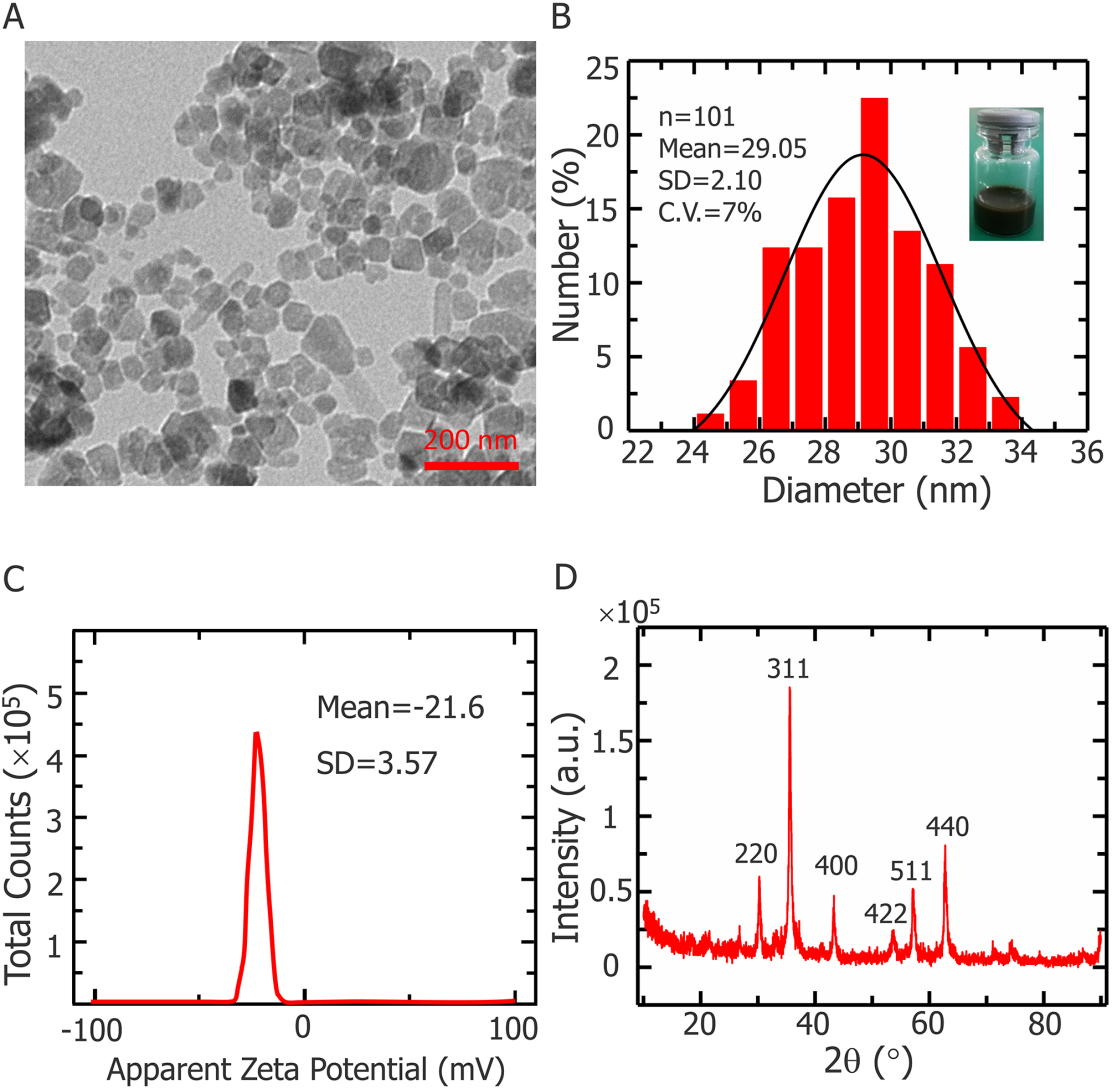
Fe_3_O_4_ NPs characterization **(A)** Representative TEM images of Fe_3_O_4_ nanoparticles. **(B)** Size distribution of Fe_3_O_4_ nanoparticles determined by dynamic light scattering at 25°C. Inset: snapshot of Fe_3_O_4_ nanoparticles dispersion with deionized water. **(C)** Apparent zeta potential of Fe_3_O_4_ nanoparticles. **(D)** X-ray diffraction pattern of Fe_3_O_4_ nanoparticles.

Figure 1 illustrates the characterization of Fe_3_O_4_ nanoparticles synthesized with a chemical coprecipitation method. The morphology of Fe_3_O_4_ nanoparticles were determined by transmission electron microscopy (TEM). Figure 1A shows that nanoparticles are uniform in size (∼25 nm) and dispersed well in aqueous solutions. Size distributions of nanoparticles appear in Figure 1B, which is measured with dynamic light scattering (DLS) at 25°C. Data for the apparent zeta potential of Fe_3_O_4_ nanoparticles are shown in Figure 1C, and the X-ray powder diffraction (XRD) patterns of the nanoparticles are shown in Figure 1D.

**Figure 2:**
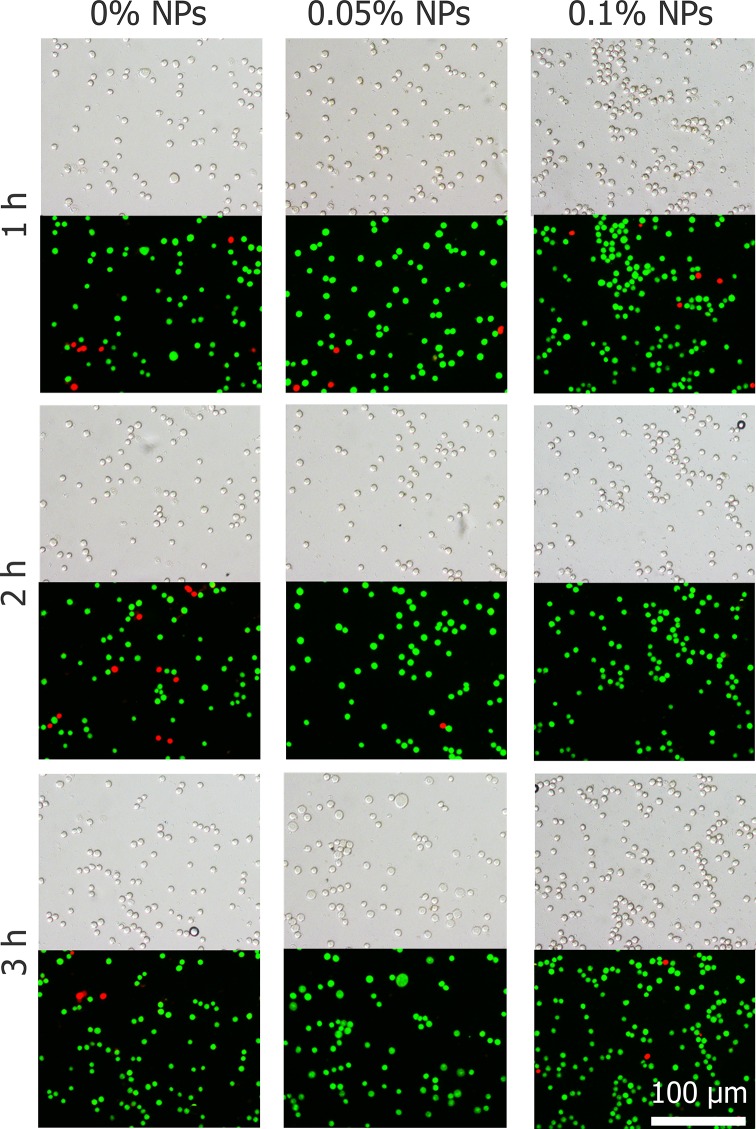
Cell morphology and viability of HepG2 cells exposed to nanoparticles after 1, 2 and 3 h

**Figure 3:**
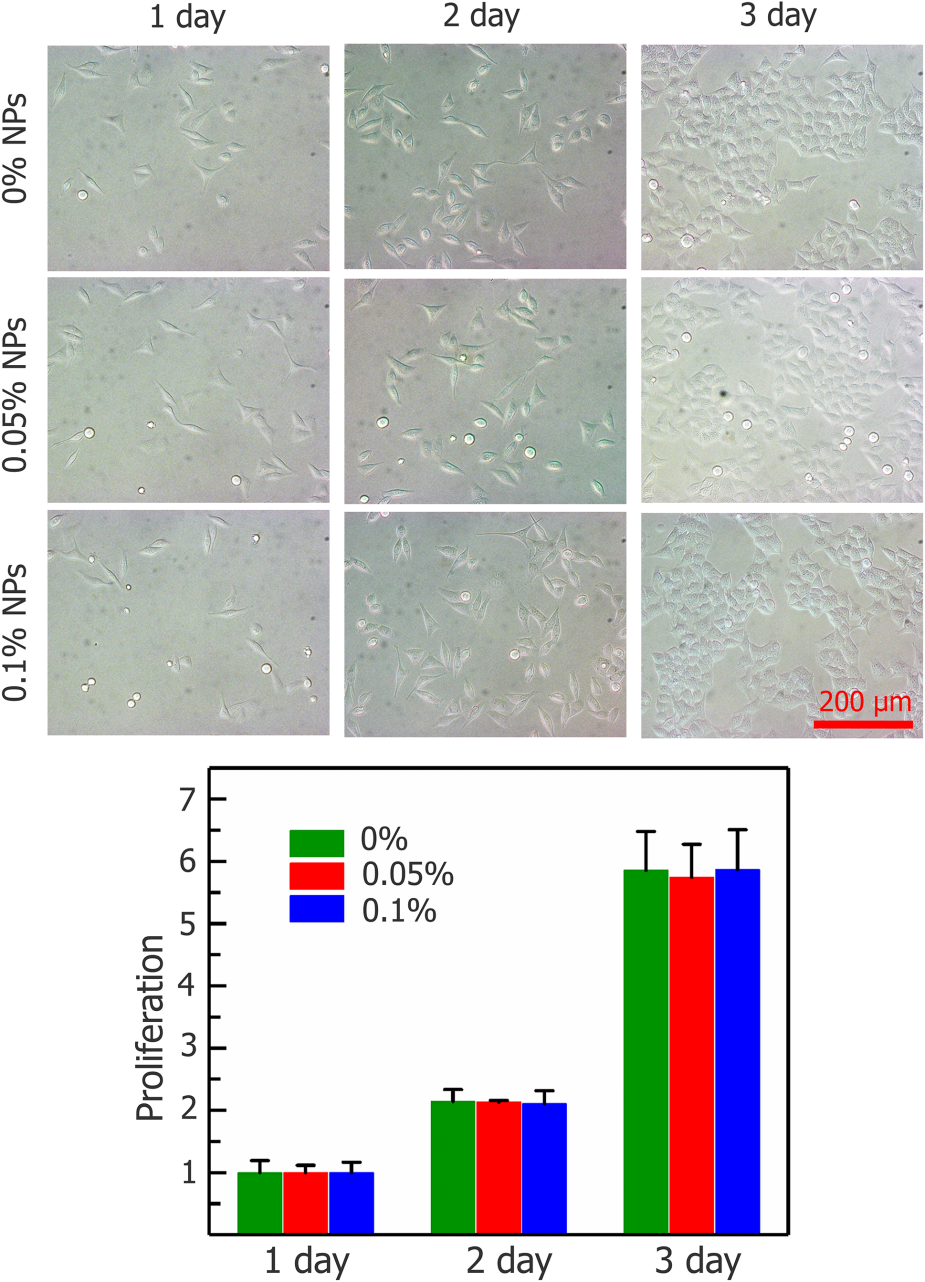
Proliferation of HepG2 cells after incubating with Fe_3_O_4_ nanoparticles (0, 0.05, and 0.1%) for 1, 2, and 3 days Data are presented means ± SD (n=5).

The effects of Fe_3_O_4_ nanoparticles on the viability of HepG2 cells are shown in Figure 2. The viabilities of the cells treated with nanoparticles after 3 hours at 37°C were more than 95%, which means that nanoparticles do not harm any activity of cells. The concentration of 0.1% (w/v) Fe_3_O_4_ nanoparticles does not affect HepG2 cell proliferation, even after 3 d of incubation shown in Figure 3.

### Cryosurgery on agarose tumor phantom

**Figure 4:**
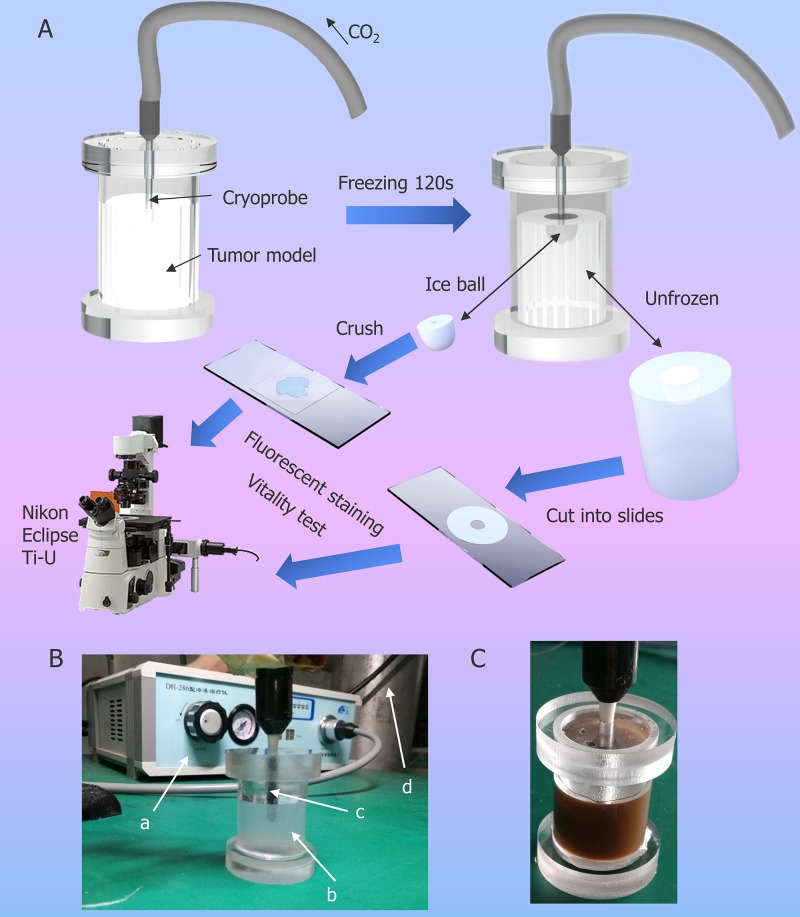
Illustration of cryosurgical device and process **(A)** Schematic diagram of the experimental procedure of cryosurgery in tumor model. **(B)** Photo of experimental set-up. a. apparatus for cryosurgery; b. agarose model with HepG2; c. cryoprobe; d. CO_2_ container. **(C)** Agarose model with HepG2 and 0.1% (w/v) NPs.

In this study, we made a new tumor phantom using agarose gel which containing HepG2 cells with Fe_3_O_4_ nanoparticles and assessed the effect of cryosurgery on cell viability in different regions (See Figure 4 for experimental setup). A flow chart of cryosurgery experiments and the illustration of experimental setup for the agarose gel study is shown in the Figure 4A and 4B respectively. The tumor model with 0.1% (w/v) concentration of Fe_3_O_4_ nanoparticles is shown in the Figure 4C.

**Figure 5:**
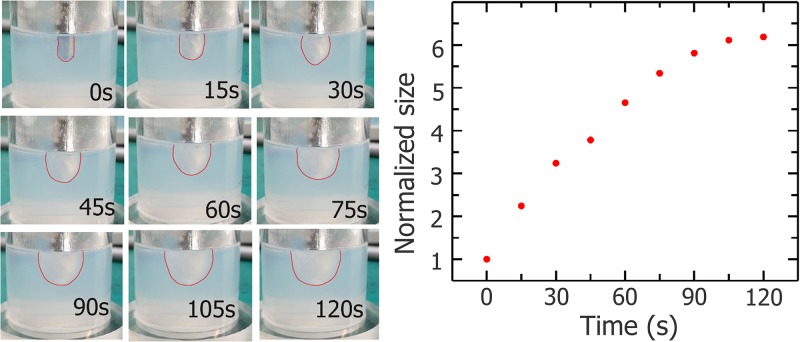
Photos of iceball (outline depicted with red lines) during cryosurgery experiment and growth curve

During the freezing process (120 s), the tissue around the cryoprobe is gradually frozen to form an iceball. As shown in Figure 5, with the freezing process continue, the ice ball enlarges and the growth rate decreases gradually.

**Figure 6:**
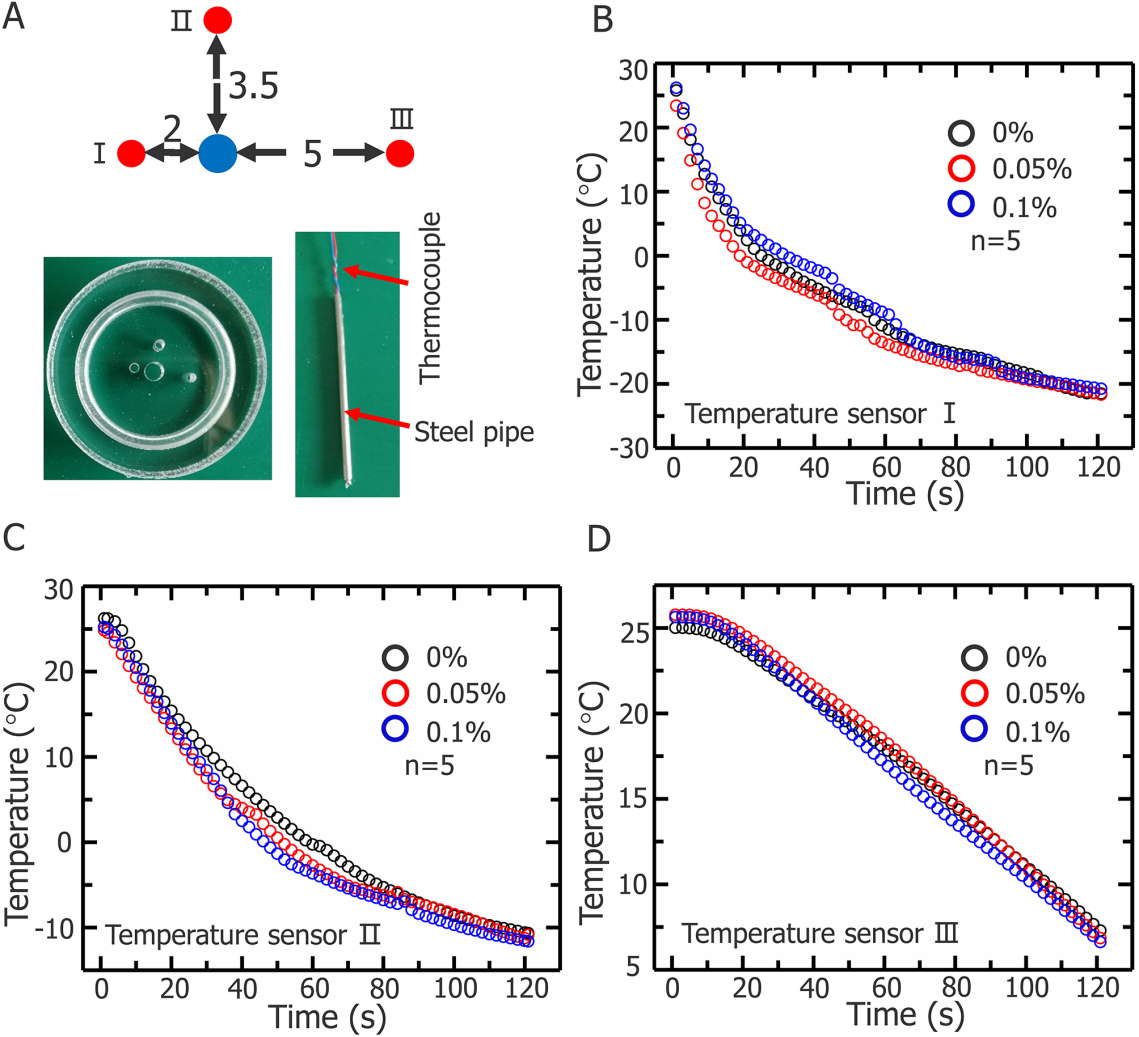
Temperature changes of three representative positions during cryosurgery **(A)** Schematics of three positions and temperature sensor (unit: mm). Temperature changes of first **(B)** second **(C)** and third points **(D)**.

Figure 6 illustrates the temperature changes during cryosurgery at three points. Thermocouple was inserted into a steel pipe (Figure 6A) to ensure that the temperature of the same point is measured for each experiment. Figure 6B, 6C and 6D shown the temperatures for all points measured.

**Figure 7:**
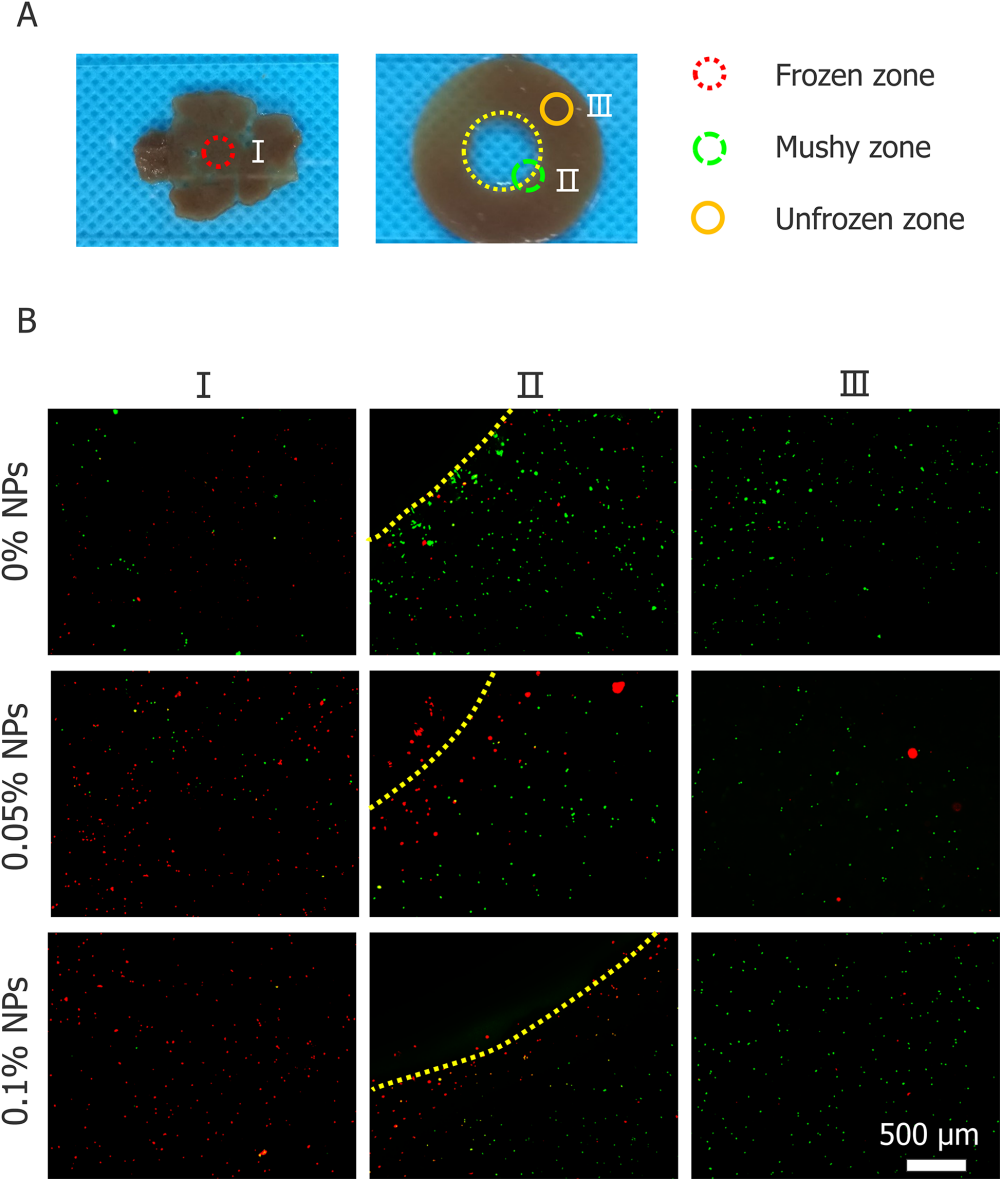
Cell viability of three regions of tumor model **(A)** Illustration of three regions. **(B)** Cell viability test for three regions treated with 0, 0.05 and 0.1% (w/v) nanoparticles (visualized with 4× Objective lens).

Figure 7 shows the cell viability in different regions after cryosurgery with different concentration of nanoparticles. Cell viability was tested in three different regions which were marked in Figure 7A. And in the frozen region of without additional nanoparticles, some cancer cells survived after cryosurgery which are well shown in Figure 7B, this may certainly lead to tumor recurrence and metastasis. When Fe_3_O_4_ nanoparticles were added to the tumor phantom, almost all cancer cells in the frozen region died. Mortality increased in proportion to nanoparticle concentration and at 0.1% (w/v), almost all cancer cells were eradicated. Unfrozen cells outside the iceball edges were not harmed. This proves that cryosurgery can control cryoinjury to surrounding healthy tissue.

### Cell-scale analysis of simulated cryosurgery by cryomicroscopy

**Figure 8:**
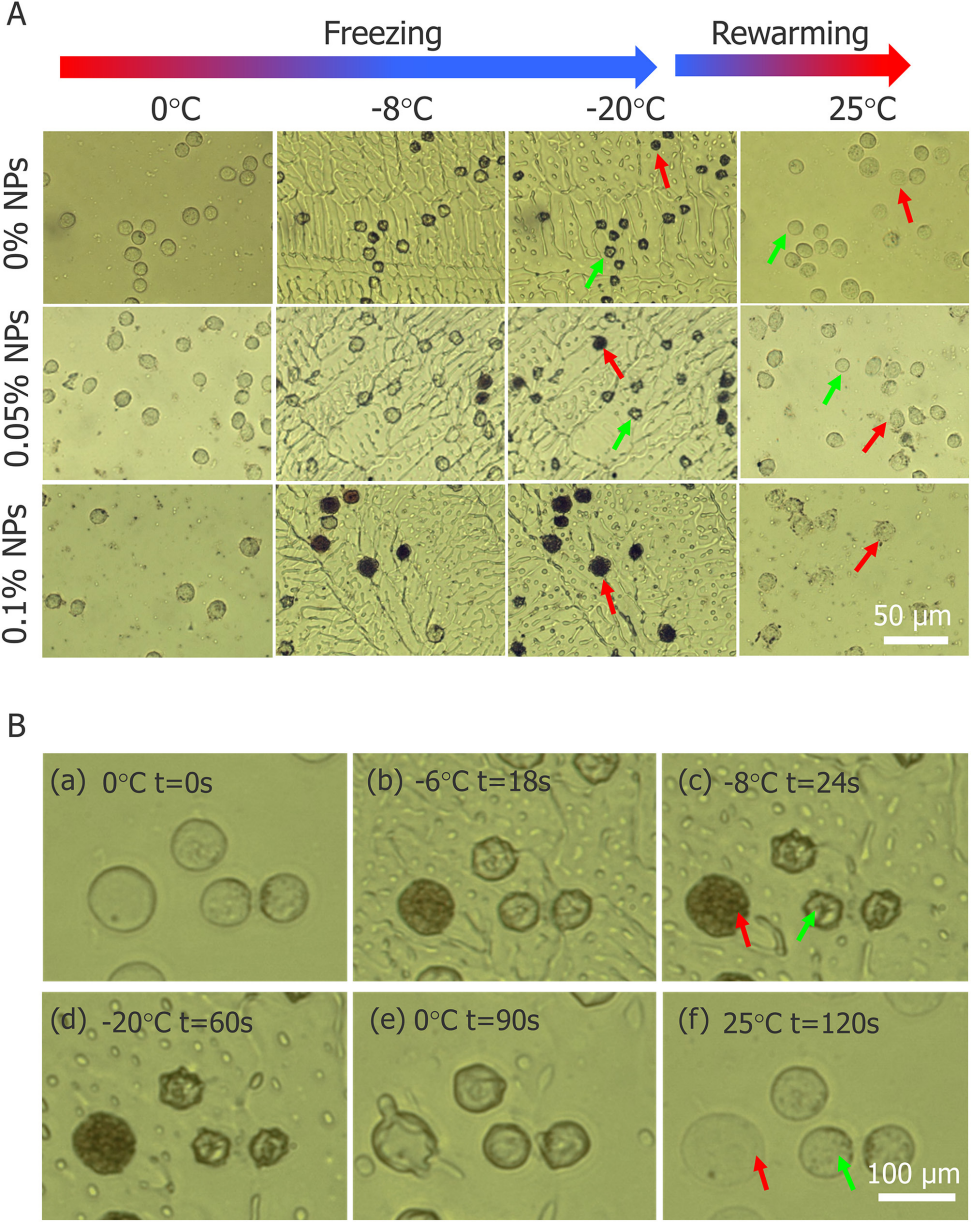
Images of HepG2 cells during freezing and thawing process in cryomicroscopic experiments **(A)** Morphology of HepG2 cells with three different concentrations of Fe_3_O_4_ nanoparticles during cryomicroscopic experiments (visualized with 20× objective lens). **(B)** Two different fates of HepG2 cells without nanoparticles during cryomicroscopic experiments (visualized with 50× objective lens). Red arrow indicates cells with IIF and green arrow indicates cells without IIF.

To understand the mechanism of how nanoparticles enhance the killing effect, we conducted cryomicroscopic experiments and monitored the variation in HepG2 cells during the cryosurgery. Figure 8 illustrates the morphology of HepG2 cells during freezing and thawing process in cryomicroscopic experiments. The results with a cooling rate of 20°C/min and a final temperature at -20°C for 2 min is shown in Figure 8A. The morphology of HepG2 cells at several temperatures during the freezing-thawing procedure is depicted. Prior to ice crystal formation (first column, Figure 8A, 0°C), almost all cell membranes remain intact. When ice crystals begin to grow (second column, Figure 8A, -8°C), some HepG2 cells turn black (red arrows), indicating formation of interior ice crystals and cell boundaries begin to shrink (green arrows) indicating dehydration. The unfrozen cellular water has a higher chemical potential than the water in the partly frozen solution outside the cell, and in response to this difference of potential, water leaves the cells, causing shrinking. When the temperature decreased to -20°C (third column, Figure 8A), the probability of IIF increased with increasing nanoparticle concentration. After thawing, some cell borders were blurred to obscurity (red arrows), suggesting that the cytomembrane was damaged, but the other cell borders remained intact (green arrows), which indicated survival.

**Figure 9:**
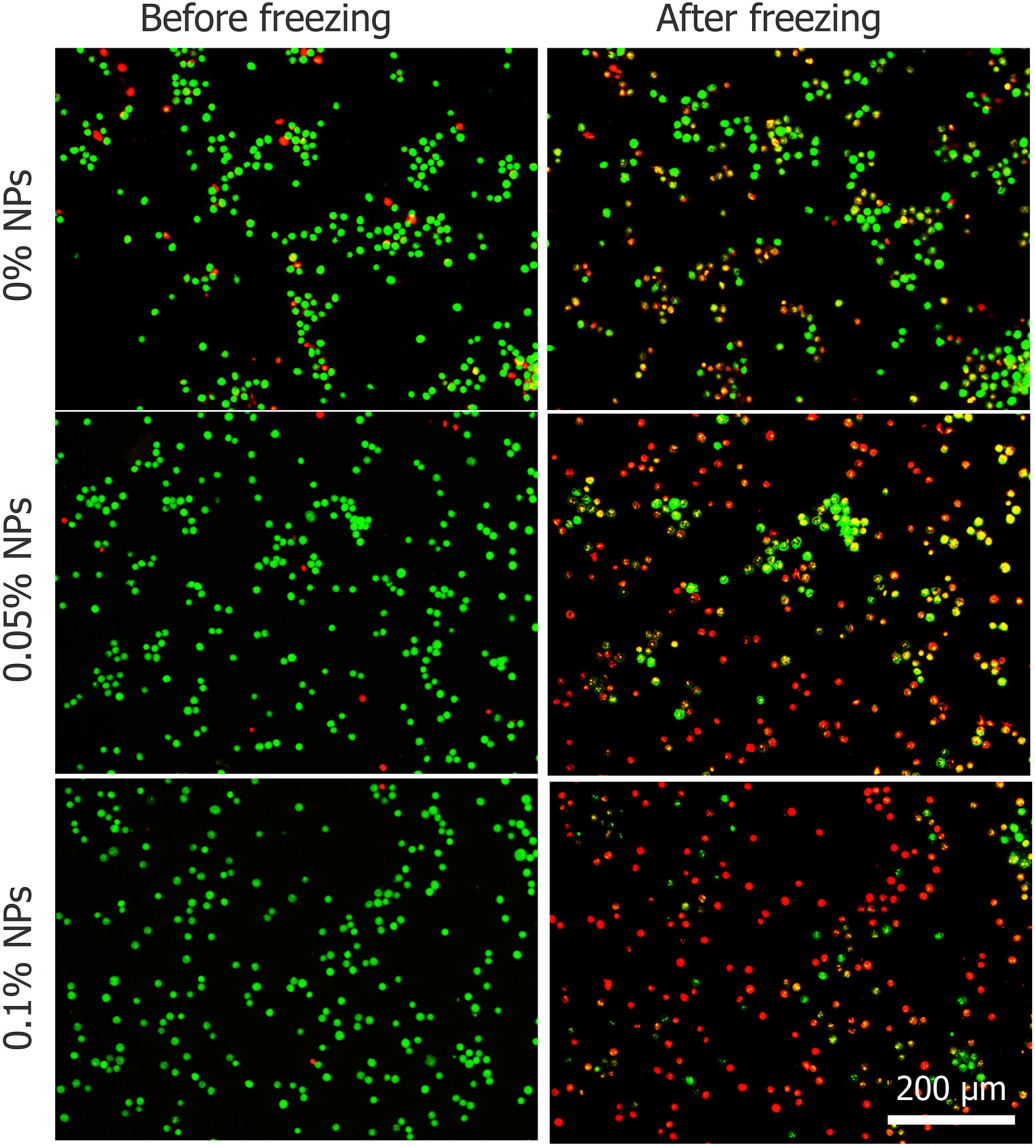
The viability of HepG2 cells before and after freezing process with three different concentrations of Fe_3_O_4_ nanoparticles

During the freezing-thawing process samples were visualized by 50× objective lens which confirmed that, cell with IIF (red arrows) had membrane destruction and cells without IIF (green arrows) retained membrane integrity (shown in Figure 8B). In addition, Figure 8B indicates that IIF destroyed the cytomembrane and caused cell death. However, HepG2 viability before and after freezing was tested with AO/EB and cell death increased with increasing Fe_3_O_4_ nanoparticles concentration shown in Figure 9.

**Figure 10:**
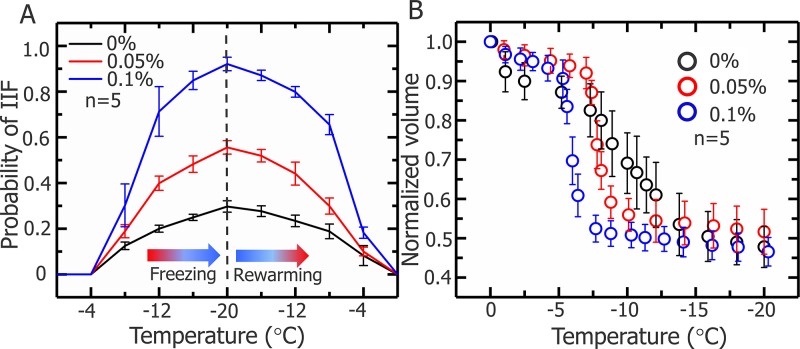
Probability of IIF in HepG2 cells and normalized cell volume during freezing process **(A)** Probability of IIF during the freezing-rewarming process corresponding to cryosurgery, HepG2 cells treated with 0, 0.05, and 0.1% (w/v) Fe_3_O_4_ nanoparticles and freezing/rewarming was achieved at rate of 20°C/min. Data are means ± SD (*n*=5). **(B)** Normalized volume of HepG2 cells treated with nanoparticles during freezing process. Data are means ± SD (*n*=5).

Figure 10 shows the effect of different concentrations of nanoparticles on probability of IIF and cell dehydration. In the 0.1% (w/v) concentration of Fe_3_O_4_ nanoparticles, the probability of IIF significantly increased more than 90%, thus facilitating the killing of tumor cells (shown in Figure 10A). Moreover, variations in cell volume have been measured to assess the effect of nanoparticles on cellular water transport, in addition nanoparticle accelerate cell dehydration is presented in the Figure 10B.

## DISCUSSION

Earlier studies demonstrated that Fe_3_O_4_ nanoparticles are biocompatible, easy washing and cost-effective nanomaterials [41–45]. Furthermore, it has been found that magnetic therapeutic agents can accumulate in the tumoral region under magnetic guidance, and hence dramatically reduce the adverse side effect on normal tissues. Fe_3_O_4_ has been extensively used for transporting a variety of drugs to the target area under a localized magnetic field [46–48]. Therefore we choice to conduct this study with self-synthesized Fe_3_O_4_ nanoparticles. Our particles are synthesized using a chemical coprecipitation method and biocompatibility of the nanoparticles was confirmed with a cell proliferation test. XRD patterns for Fe_3_O_4_ nanoparticles powder (Figure 1D) shows that all peaks are quite identical to Fe_3_O_4_ (JCPDS No. 82-1533), indicating that the sample has a cubic crystal form [42, 49, 50], furthermore, no characteristic peaks of impurities were observed. The mean particle diameter can be calculated from the XRD pattern according to the Scherrer formula [51]. The value of diameter obtained by x-ray diffraction is 26.82 nm, smaller than the result from the DSL test. The measured diameter in the DLS test is not the physical diameter, but the hydrodynamic diameter [52]. And as light scattering can be approximated as a Rayleight type of scattering, then average diameter derived from the DLS test should be considered an average of the larger nanoparticles [52, 53]. Thus DLS data are consistent with XRD results, considering aforementioned facts. During the experiment, most of the nanoparticles were outside the cells, since the duration of the experiment was short (less than ten minutes), As previous experiments have shown that, large amount of Fe_3_O_4_ nanoparticle takes several hours to immersed into the cells [54, 55]. Previously, an *in vivo* cryosurgery experiment were performed by Liu's group with MgO nanoparticles size of approximately 50 nm. They injected MgO nanoparticles with physiological saline solution of a mass fraction of 5% into the rabbit tissue for controlling the shape of the frozen region during surgery [26]. The Fe_3_O_4_ nanoparticles we introduced in this study have a diameter of approximately 27 nm, the smaller size ensures that the nanoparticles can move easily within the tumor tissue. The maximum concentration of nanoparticles in our work is 0.1% (w/v), which is easy to initialize into the phantom and also possible to achieve this amount of nanoparticles *in vivo* experiments.

The present cryosurgery experiment in an agarose model with HepG2 cells demonstrates that the Fe_3_O_4_ nanoparticles could improve the killing efficiency and the range of killing is extended to the entire iceball, subsequently, the cryomicroscopic experiments verified the inner mechanism of enhance killing of tumor cells through addition of Fe_3_O_4_ nanoparticles. The clinical monitoring methods for cryosurgery (CT, ultrasound and X-ray) are almost based on the visualization of the iceball, therefore the assessment of precise killing of all tumor cell was subjective. Depending on the type of tumor cells, the traditional method of assessing the effect of cryosurgery is -20°C or -40°C which considered as lethal temperature [32–35]. That means the tumor cells will be killed when the temperature reaches -20°C or -40°C, however, for a normal cryosurgery process, the effective killing region (the critical isothermal surface) is always smaller than the iceball and the temperature distribution inside the iceball is invisible during the cryosurgery. As a result, the end of the cryosurgery process can only be judged by the surgeons according their experience. The subjective judgement is one of the main reasons for poor estimation of tumor ablation and a high probability of recurrence and metastasis associate with cryosurgery, which results in uncertainty in the final outcome of the cryosurgery. In this study, we introduced an agarose tumor model with uniform distribution of HepG2 cells, and it is more effective and reliable to evaluate the killing efficiency through cell viability compared with previous model experiments which evaluate the effect of cryosurgery through the temperature distribution [18, 26, 34]. We noted that some cells were not killed in the frozen region when nanoparticles were not used, especially at the edges of the iceball (Figure 7B). With the help of nanoparticles, the killing effect of cryosurgery was enhanced and there was no additional damage to the surrounding unfrozen tissue (Figure 7B), which means we can precisely control the scope of the killing to reduce the damage to normal cells. In addition, being different from the previous optimization studies, we can expand the effective killing region to the entire iceball with the aid of nanoparticles, and thus to greatly decrease the difficulty in precise judgement of the end for cryosurgery only by using the commonly used clinical imaging methods. Moreover, cryosurgery-treated cells with 0.1% (w/v) nanoparticles are completely dead, which verifies the outcome of entire tumor destructions, therefore we have not test higher concentration of nanoparticles. The tendency of temperature change has a similar correspondence with the change of iceball volume, besides, the temperature changes of cryosurgery in three different situations are almost identical, which means that the introduction of 0.1% (w/v) Fe_3_O_4_ nanoparticles causes no significant thermal effects (Figure 6B).

To explore the mechanism of Fe_3_O_4_ nanoparticles enhanced the treatment effect, a cryomicroscope was used to monitor the variations in IIF and osmotic responses of HepG2 cells with/without Fe_3_O_4_ nanoparticles. Fe_3_O_4_ nanoparticles were previously reported to enhance ice crystal formation and improve killing of breast cancer cells [23]. In addition, investigated on the intracellular ice formation during freezing process of MCF-7 cancer cells has been performed [56]. Despite some studies were performed with cryomicroscope to explore the mechanism of cell death during freezing, they did not combine the cryomicroscopic experiments with cryosurgery investigation, thus cannot reflect the process of cancer cells suffering from cryosurgery aspects. The cooling rate for cryomicroscopic experiments was 20°C/min, which is consistent with the temperature collected in cryosurgery experiments. The introduction of nanoparticles certainly catalyzes the growth of extracellular ice crystals, while the entire mechanism of successful IIF is still debated. There are many suppositions that have been presented and verified by experiments. One of them is, Intracellular ice formation (IIF) may be catalyzed by the plasma membrane via the effects of the external ice formation on the plasma membrane, this assumption is well known as surface-catalyzed nucleation (SCN) [57, 58]. And other one is, Extracellular ice crystals may enter the cells through the micropores on the cell membrane, and the involvement of these tiny ice crystals promotes the formation of intracellular ice crystals [59–61]. Based on aforementioned theories, we can expect that the introduction of nanoparticles catalyzes the formation of ice crystals inside the cell membranes (Figure 8A and Figure 10A). In this study, many cell membranes exhibited a complete structure after a traditional cryosurgery (without nanoparticles) and these cells may cause tumor recurrence (Figure 8A). During the traditional cryosurgery (without nanoparticle added), intracellular ice crystals can cause irreversible damage to the cell membrane which insures the killings of cells, however some of the dehydrated cells may regain water during rewarming process and retain viability (Figure 8B), which means intracellular ice crystals is a lethal factor compared to cell dehydration. Moreover, the introduction of nanoparticles may accelerate extracellular ice growth prominently, and this accelerated extracellular ice growth, causing more rapid cell shrinkage (Figure 10B), which ultimately produce membrane destruction and protein damage. As Figure 9 shows that, once the nanoparticle introduces, cell mortality is higher than the probability of IIF and Figure 10A eventually verifies that cells die due to both phenomena (dehydration and IIF). Furthermore, fluorescent images from the cryomicroscopic experiment (Figure 9) are consistent with those taken from cryosurgery (Figure 7B).

In this study, we used an ideal tumor model without vascular networks. The process of neovascularization is generally characterized for the tumor larger than a few millimeters, which results from the fact that tumors are often situated near some large blood vessels [62, 63], and also some tumor types malignancies, such as pancreatic tumors are closely situated with aorta and other major vessels [64]. Unlike *in vivo* tumors, the tumor model does not take blood flow and metabolism into account while the convective effect of the blood flow and metabolism may significantly heat the surrounding cold tissues frozen by the cryoprobe [65]. Moreover, different tumor cells have different lethal temperatures [38], therefore the results may be cell type dependent. In addition, further experiments on size effect of Fe_3_O_4_ nanoparticles on the viability of HepG2 cells under the same conditions need to be performed for verification of particle size effects on IIF and dehydration.

In summary, we successfully developed a novel method for high-precision killing cells during cryosurgery with Fe_3_O_4_-nanoparticle enhanced intracellular ice formation and cell dehydration. It was found that the introduction of the self-synthesized Fe_3_O_4_ nanoparticles can significantly improve the killing of tumor cells during cryosurgery, and the range of killing was extended to the entire iceball. The potential mechanism is further revealed by the cryo-microscopic experiments, which verifies the presence of Fe_3_O_4_ nanoparticles can significantly enhance the probability of intracellular ice formation and the cell dehydration during freezing and thus to promote the killing of tumor cells. These findings may further promote the widespread clinical application of modern cryosurgery.

## MATERIALS AND METHODS

### Synthesis and characterization of Fe_3_O_4_ nanoparticles

Fe_3_O_4_ nanoparticles were synthesized with a modified chemical coprecipitation method using FeCl_2_·4H_2_O and FeCl_3_·6H_2_O [66]. First, 5 ml of ammonia (Kelong Chemical Reagents Co. Chengdu, China) and 2 ml of hydrazine (Aladdin Ltd., Shanghai, China) were mixed and diluted with deionized water to 50 ml. Second, 1 g of FeCl_2_·4H_2_O (Aladdin Ltd., Shanghai, China) and 2.7 g of FeCl_3_·6H_2_O (Aladdin Ltd., Shanghai, China) were mixed and diluted with deionized water 20 ml, and the mixture was dropwise added into the 50 ml solution mentioned above and stirred at 90°C for 30 min. Then, 10 ml 40% (w/v) citric acid (Sangon Biotech Co. Ltd., Shanghai, China) was added to this mixture solution and stirred at 90°C for 1.5 h. Finally, the products were washed alternately with water and acetone twice. Fe_3_O_4_ nanoparticles were dried and stored at room temperature for future use.

Transmission electron microscopy (TEM, JEM-2011, Hitachi, Ltd., Tokyo, Japan) was used to examine Fe_3_O_4_ nanoparticle morphology at an accelerating voltage of 100 kV. Dynamic light scattering (DLS) (DynaPro-MS800, Wyatt Technology, Santa Barbara, CA) was used to evaluate Fe_3_O_4_ nanoparticle size. The apparent zeta potential was measured with a Malvern zetasizer (Nano ZS90, Malvern instruments Ltd., UK). X-ray powder diffraction (XRD) patterns of samples were confirmed at room temperature with a diffractometer (TTR III, Rigaku Co., Tokyo, Japan). Finally, data were collected from 10 ° to 90 ° with a resolution of 0.02 °. Average crystallite size was estimated using the Scherrer equation:

$$D=\frac{K\lambda }{B\cos\theta }$$ (1)

The diffraction peak with the highest intensity was selected for the calculation, where K is a Scherrer constant, about 0.89, *λ* is the X-ray wavelength (0.154056 nm), B is the width of the XRD peak at half height and *θ* is the diffraction angle.

### Cell culture

HepG2 cells were cultured in Dulbecco's modified Eagle's medium (DMEM), containing 10% (v/v) fetal bovine serum (FBS, Hyclone, Thermo Fisher Scientific, Inc., Waltham, MA) and 1% (v/v) penicillin-streptomycin liquid (Hyclone, Thermo Fisher Scientific, Inc., Waltham, MA). Trypsin was obtained from Biosharp (Biosharp Co., China). The medium was changed daily until reaching 80–90% confluency, and then the cells were detached with 0.25% (v/v) trypsin-EDTA (Gibco, USA), centrifuged at 100 x g for 5 min, and resuspended in culture medium for passaging and/or further experimental use.

### Cytotoxicity of Fe_3_O_4_ nanoparticles

To assess the Fe_3_O_4_ nanoparticle cytotoxicity and cell proliferation in HepG2 cells [67–69], centrifuged cells were resuspended using media containing different concentrations of nanoparticles, and cell viability was tested after 1, 2 and 3 h at 37°C. Subsequently, cell proliferation experiments were performed. Centrifuged cells were resuspended in 96-well plates in 100 μl medium, and a CCK-8 reagent Kit (Dojindo Inc., Kumamoto, Japan) was used to measure cell proliferation. The cells were incubated overnight then the medium were removed and the cells were treated with medium containing 0, 0.05% and 0.1% (w/v) Fe_3_O_4_ NPs in a 37°C, 5% CO_2_ humidified incubator for further culture for up to 3 days. After the medium with NPs were removed per well, 100 μl complete medium and 10 μl cell count kit-8 reagent were added per well and the plate was incubated for 4 h in incubator and then the absorbance was measured at 450 nm using an enzyme-linked immunosorbent assay (ELISA) plate reader (Diagnostics Pasteur, Marne la Coquette, France). The proliferation capacity was quantitatively assessed as the relative cell number on days 2 and 3 to that on day 1.

### Cryosurgery on agarose tumor phantom

An aqueous solution containing 2.5% (w/v) agarose (Biosharp Co., China) was prepared using a magnetic stirrer (MS-H-Pro+, SCILOGEX, LLC, USA), and the temperature was set to 70°C. When the solution became colorless and transparent, the rotor was removed, and the solution was allowed to cool at room temperature. Then, 1 ml of cell suspension with/without Fe_3_O_4_ nanoparticles was added to the solution when the temperature reached 37°C. The solution was gently stirred with a glass rod for one min to ensure that cells were evenly dispersed in the solution. The tumor model container was Plexiglas and after the tumor solidified, a cryoprobe (DH-286, DaHai mechanical and electrical equipment factory, JiangSu, China) was inserted into the center of the model and fixed with a clamp to ensure a constant cryoprobe position. After two min of cryosurgery, the tumor model was divided into two parts, an iceball and an unfrozen portion. The iceball was placed on a slide and crushed using a coverslip, and the unfrozen area was cut into 1 mm thickness using a scalpel. Another modified container was used for temperature experiments. The container had three holes in the lid for inserting the temperature sensor, which was made by inserting the thermocouple (T type) into a steel pipe (Figure 6A). During freezing, the temperature was collected per second with an Agilent data logger (34970A, agilient, Agilent Technologies, Inc. USA). The samples were taken from the relative middle position of cryoprobe and the thermocouple measures the temperature of same vertical dimensions of cryoprobe. After the freezing, when cryoprobe was removed, the iceball were attached to the cryoprobe and it separate from the unfrozen part of gel, the iceball was crushed with coverslips and the viability of HepG2 cells after cryosurgery were evaluated using an acridine orange/ethidium bromide (AO/EB) staining kit (KeyGen BioTECH Co., Ltd., China). A fluorescent staining solution (AO:EB = 1:1) was add to the treated tumor model and fluorescent images of cells were taken using a DS-Ri1 (Nikon, Tokyo, Japan) camera and 4× objective equipped on a fluorescent inverted microscope (Nikon Eclipse Ti-U, Tokyo, Japan).

### Cell-scale analysis of simulated cryosurgery by cryomicroscopy

The cryomicroscope consists of a cryostage (HCS302GXY, Instec, Inc. USA), liquid nitrogen pump (INSTEC, Inc. USA) and temperature controllers (MK2000, Instec, Inc. USA). A small drop (4 μl) of cell suspension with different concentrations of nanoparticles was pipetted onto the center of a round coverslip and another coverslip was placed on top. When the coverslip was pressed, cells flowed, indicating that the solution between the coverslips was greater than the diameter of cells and the cells were not compressed. HepG2 cells were cooled form 25°C to -20°C at rate of 20°C/min, and then held for 1 min at -20°C. Finally, cells were rewarmed to 25°C at rate of 40°C/min. Data for transmembrane water transport and IIF were recorded with CCD (MicroPublisher 5.0 RTV, Survey, BC, Canada) and 20×, 50× long working distance objectives. The probability of IIF was equal to the number of cells that underwent IIF at a certain temperature divided by total number of cells in the observation [23].

The same experiment was repeated for around 100 times, 20–100 cells were observed for each run. Data were then randomly divided into five groups (500 cells/group). Three representative experiments of three conditions were selected, and each had at least 20 temperature points. Cell volume changes at each temperature point were measured and data were normalized according to the starting volume. Cell viability was tested before and after freezing. The total pixel area inside a cell was tallied then converted to square micrometers. The obtained cell area was then used to calculate the equivalent radius of the cell. Cell volume was further estimated using the equivalent cell radius.
